# Prehypertension and Hypertension among Schoolchildren in Brazzaville, Congo

**DOI:** 10.1155/2014/803690

**Published:** 2014-05-20

**Authors:** Bertrand Fikahem Ellenga Mbolla, Annie Rachel Okoko, Jean Robert Mabiala Babela, Gaston Ekouya Bowassa, Thierry Raoul Gombet, Suzy-Gisèle Kimbally-Kaky, Benjamin Longo-Mbenza

**Affiliations:** ^1^Department of Cardiology, University Hospital of Brazzaville, BP 32, Brazzaville, Congo; ^2^Department of Medecine, Faculty of Health Science, Marien Ngouabi University, BP 2672, Brazzaville, Congo; ^3^Department of Pediatrics, University Hospital of Brazzaville, BP 32, Brazzaville, Congo; ^4^Faculty of Health Science,Walter Sisulu University, Mthatha, P.O. Box 5117, Eastern Cape, South Africa

## Abstract

*Background.* To determine the prevalence and associated factors of prehypertension (pre-HT) and hypertension (HT) in schoolchildren at Brazzaville (Congo). *Methods.* This cross-sectional study was conducted from March to May 2011 in five representative urban schools in Brazzaville. American Pediatric Society's definition of pre-HT and HT was used. The measurement of blood pressure was obtained using auscultator method. Univariable and multivariable analyses were performed to establish associations between blood pressure levels and sociobiographical factors. *Results.* 603 children were included. The mean age was 11.8 ± 3.6 years (range 5–18 years). The prevalence of pre-HT was 20.7% (*n* = 125). Factors associated with pre-HT were secondary school (*P* = 0.02), private schools (*P* < 0.004), migrants (*P* = 0.03), the obese (*P* = 0.004), high socioeconomic level (*P* < 0.01), and overweight (*P* = 0.02). In logistic regression, the independent determinants of pre-HT were secondary school (*P* = 0.0001), migration (*P* = 0.04), obesity (*P* = 0.004), and overweight (*P* = 0.01). The prevalence of HT was 10.1% (*n* = 61) during the first screening and 3.3% (*n* = 20) in second screening. The independent determinants of HT were obesity (*P* = 0.0001) and overweight (*P* = 0.0001). *Conclusion.* Pre-HT and HT are emerging as a mass problem in Congolese schoolchildren with urban migration and overweight/obesity to be controlled and prevented.

## 1. Introduction

Hypertension (HT) in children is a rare concern whereas HT in adults is a global public health problem, according to World Health Organization (WHO) [1]. The prevalence of HT is reported in 4 to 15% children worldwide [2], with increasing trends during recent decades [3, 4]. This increase is attributable to particular stress of mega cities in industrialized countries and lifestyle and diet whose impact on body size is well established. In sub-Saharan Africa (SSA) which is facing epidemiological transition, the prevalence of HT in child varies by regions: 4.9% in schools from Sudan [5] and 29.4% from South Africa [6]. However, no data on HT and prehypertension (pre-HT) is available in schoolchildren from Republic of Congo.

The objectives of this study were to determine the prevalence of pre-HT and HT and to identify its contributing factors, which would establish an effective prevention program starting from a young age in Brazzaville, Congo.

## 2. Methods

### 2.1. Type, Period, and Materials

This cross-sectional study was conducted from March to May 2011 in five schools of Brazzaville, divided in two public schools and three private schools. These institutions were selected by cluster sampling (cluster was defined as all establishments of a school district), after a random selection of 1/10 was carried out among 219 schools from the city of Brazzaville at the time of the survey. At the time of the study, this town had three school districts, according to the division made by the Department of Elementary and Secondary Education of Congo. The criteria of inclusion approach were consistent with that of Kimbally-kaky et al. [7]. Thus, the calculated sample size was 600 + 6 (10% potential missing) = 606 eligible schoolchildren.

The parents, schoolchildren, and schools responsible authorities were sensitized about the aim, the significance, and the timetable for the study. Ethical issues were taken into account according to the Helsinki Declaration. The learners were interviewed and examined by investigators after prior training. Systolic blood pressure (SBP) and diastolic blood pressure (DBP) were recorded three times according to the American Pediatrics Society recommendations, using auscultatory method [8] by aneroïde sphygmomanometer (Spengler, France). The average of three taken pressures was retained.

### 2.2. Variables

The analyzed variables were age (years), sex, weight (kg), height (cm), and body mass index (BMI) in kg/m^2^, malnutrition, overweight, obesity, adolescence, migration, number of siblings, promiscuity, school sector activity (primary, secondary, public, and private), high socioeconomic level, SBP in mm Hg, DBP in mm Hg, pulse pressure (PP) in mm Hg, HT, and pre-HT.

### 2.3. Definitions

Adolescence was defined by WHO. Migration is defined by expatriation in Brazzaville since three years. Promiscuity was considered the presence of at least three children in the bedroom. The high socioeconomic level was defined according to parental occupation, namely, civil servants, traders, or a higher income than twice the minimum salary in Congo. Malnutrition was defined for BMI < 5th percentile of WHO reference, overweight for BMI between 85th and 95th percentile, and obesity for BMI > 95th percentile [9]. Pre-HT was defined for SBP and DBP between 85th and 95th percentile of American Pediatrics Association curves reference [10]. HT was defined by SBP and DBP ≥ 95th percentile of American Pediatrics Association curves reference [10].

### 2.4. Statistical Analysis

Data were expressed as proportions for categorical variables and as mean ± standard deviation for continuous variables. In univariate analysis, Student* t*-test, chi-square test, and odds ratio (OR) with 95% confidence interval (CI) were used for comparisons of means and proportions and associations, respectively. In multivariate analysis, after adjusting for confounding factors, logistic regression models identified independent determinants of pre-HT and HT. The criterion for statistical significance was *P* value < 0.05. All analyses were performed using SPSS software version 10.0 for Windows (SPSS Inc., Chicago, IL, USA).

## 3. Results

In total, 603 learners (response rate of 99.5%) participated with 325 girls (54%) and 278 boys (46%) having similar (*P* = 0.08) age (mean 11.8 ± 3.6 years, range 5–18 years). Epidemiological characteristics were similar (*P* > 0.05) between girls and boys (Table 1).

Table 2 presents comparisons of mean levels of weight, height, BMI, SBP, DBP, and PP between girls and boys: similar (*P* > 0.05) levels of height, SBP, DBP, and PP between girls and boys, whereas girls were significantly (*P* < 0.05) heavier (Height and BMI) than boys. Malnutrition, overweight, and obesity were reported in 62 (10.2%), 47 (7.8%), and 25 (4.1%) learners, respectively.

The prevalence of pre-HT was 20.7% out of all schoolchildren (*n* = 105), 24.3% in girls (*n* = 79), and 16.6% in boys (*n* = 46). The mean age was 13 ± 3.3 years (range 5–18 years) for pre-HT children, versus, 11.5 ± 3.7 years (range 5–18 years) for children without pre-HT (*P* < 0.0001). The mean BMI in pre-HT children was 19.5 ± 3.5 kg/m^2^ (range 12.8 to 30.1 kg/m^2^) versus, 17.3 ± 3 kg/m^2^ (range: 12.5 to 29.3 kg/m^2^) for learners without pre-HT (*P* < 0.01). The univariate associated factors of pre-HT were the private school, secondary school, obesity, overweight, and high socioeconomic level (Table 3). In logistic regression, the independent determinants of pre-HT were secondary school (*P* = 0.0001), migration (*P* = 0.04), obesity (*P* = 0.004), and overweight (*P* = 0.01) (Table 4).

The prevalence of HT in the first screening was 10.1% (*n* = 61 cases) and 3.3% (*n* = 20) in the second screening. There were 12 girls and 8 boys and the mean age of HT children was 12.8 ± 2.6 years versus 11.8 ± 3.7 years for children without HT (*P* > 0.05). The BMI of children who had HT was 20.3 ± 4.4 kg/m^2^ versus 17.7 ± 3 kg/m2 for children without HT (*P* < 0.001). Only overweight and obesity had a positive and significant univariate association with HT (Table 5). In logistic regression, the independent determinants of HT (Table 6) were obesity (*P* = 0.0001) and overweight (*P* = 0.0001).

## 4. Discussion

This study reported the burden and contributing factors of pre-HT and HT in a sample of schoolchildren in Brazzaville, Congo. Rigorous sampling criteria of this study and that of other authors [10] demonstrated high rates of high blood pressure.

The prevalence of pre-HT was 20.7% associated with several factors in the present survey. These contributing factors included private school, secondary school, migrant children, children belonging to a family of high socioeconomic status, children with obesity and overweight, and adolescents. Prevalence of pre-HT varies by puberty, country, and social environment (degree of urbanization). Low prevalences of pre-HT are reported 4.9% by Salman et al. in Sudan in schools [5], 5.7% by Abolfotouh et al. in Egypt for adolescents in schools [11], 7.6% by Salvadori et al. in Canada [12], and 8.6% by Kemp et al. in rural areas of South Africa [6]. Intermediate prevalences of pre-HT are observed 12.3% by Sharma et al. in schools of India [13] and 15% by Guo et al. in rural areas of China [14]. However, higher rates of pre-HT were reported by this study and estimated 20.7% and similar with 22.2% and 25% among Nigerian adolescents from rural and urban areas, respectively [15].

Agyemang et al. in Ghana found that the level of blood pressure increased with age, and this was more pronounced in urban areas [16]. These facts were also reported by Paradis et al. in USA, with a prevalence of 12% for children aged 9 years, 22% for children aged 13 years, and 30% for children aged 16 years ago [17]. As remote areas, however, aging was not associated with increasing blood pressure among these Congolese schoolchildren.

The most important determinants of pre-HT in this survey were migration, obesity, and overweight. The literature reports much higher prevalence of pre-HT children with overweight or obesity [6, 18, 19]. Moreover, after migration from rural areas toward town of Brazzaville, urbanization and westernization are driving both epidemiological transition and nutrition transition characterized by changes in lifestyle and dietary habits. Thus, physical inactivity, excessive intake of salt, refined sugar, and polysaturated fats increase the risk of overweight/obesity and cardiovascular risk among children [20, 21]. But these factors are difficult to evaluate in our context. Other risk factors were found. These include children in secondary schools, private schools, and children with high socioeconomic level. Indeed, the majority of high school students are adolescents, in which a high prevalence of pre-HT is described by other authors [4, 15, 17, 22, 23]. In this study, the HT was found in 10% of children in Brazzaville, Congo. It is reported that repeated blood pressure measurements and screening may reduce prevalence of HT in some data from the literature [24], but not in the present study. The prevalence of HT varies by the country of residence and age [24]. The prevalence of HT is continuously increasing in developing countries as shown by Chiolero et al. in the Seychelles: 6.9% of children in 2004 and 7.8% in 2006 [3]. In Africa, low prevalences of HT were reported as 4.9% in Sudan [5] and 4% in Egypt [11]. On the other hand, the rate of 10.1% hypertensive black Bantu Congolese children in this study is intermediate between those from non-Bantu Sudanese and Egyptian children [5, 11] and that of 29.4% black Bantu South African children [6]. These differences are related to standards of developing or emerging economies, overweight/obesity [17–19], and ethnic/genetic factors [3, 22].

Disparities were also found outside of Africa, such as 1.08% in Argentina [25], 7.4% in Canada [12], and 19.6% in Greece [26], highlighting not only the level of development, but also the impact of policies to prevent these countries [4]. Variations in prevalence have been reported within the same country. This is the case of China, where the prevalence of 9.8% to 20.2% was observed from different regions [14, 27]. These gaps are certainly explained by differences of development between regions of high growth in this country. On the other hand, in India, the prevalence appears to be more constant, which Genovesi et al. reported as 5.2% [28], Sharma et al. as 5.9% [13], and Buch et al. as 6.48% [29]. Indeed, in this country, although the economic emergence is initiated, the divisions are still significant and the social level is still low, with non-westernized eating habits [13].

Unlike pre-HT only overweight and obesity were risk factors of HT in our study. Indeed, it is both classic factors of HT in children in various parts of the world [17–19].

## 5. Conclusion

Both prevalences of pre-HT and HT are recognized as a public health problem in schoolchildren from Brazzaville, Congo. The most important contributing factors for pre-HT and HT are migration, overweight, and obesity. Prevention and control of the consequences of lack of education are needed urgently in Congolese children.

## Figures and Tables

**Table 1:** Epidemiological characteristics of the children studied.

	All (*n* = 603)	Girls (*n* = 325)	Boys (*n* = 278)	*P* value
Adolescents	308 (51)	192 (59)	116 (41.7)	<0.05
Migration	67 (11)	40 (12.3)	27 (9.7)	NS
Number of siblings	4 ± 1.8	4 ± 1.8	3.9 ± 1.9	NS
Promiscuity	275 (45.6)	150 (46.2)	125 (45)	NS
Primary school	315 (52.2)	155 (47.7)	160 (57.6)	NS
Secondary school	288 (47.8)	170 (52.3)	118 (42.4)	NS
Private school	319 (53)	175 (53.8)	144 (51.8)	NS
Public school	284 (47)	150 (46.2)	134 (48.2)	NS
HSL*	360 (60)	196 (60.7)	164 (59)	NS
Orphans	38 (6.3)	25 (7.7)	13 (4.7)	NS

Data are mean ± standard deviation or number (%).

*HSL: high socioeconomic level.

**Table 2:** Clinical characteristics of the subjects studied.

	All	Girls	Boys	*P* value
Weight (Kg)	39.2 ± 14.8	40.5 ± 14	37.7 ± 15.5	0.01
Height (cm)	145 ± 18.8	146.1 ± 16	144 ± 20.7	NS*
BMI (kg/m^2^)^†^	17.8 ± 3.2	18.3 ± 3.3	17.2 ± 2.3	0.001
SBP (mm Hg)^‡^	112.8 ± 13	114.5 ± 12.7	110.7 ± 13	0.001
DBP (mm Hg)^§^	73.6 ± 8.8	74.5 ± 9	72.7 ± 8.6	NS
PP (mm Hg)^#^	39 ± 9	40.2 ± 8.6	38 ± 9.2	NS

Data are mean ± standard deviation.

*NS: not significant (*P* > 0.05), ^†^BMI: body mass index; ^‡^SBP: systolic blood pressure, ^§^DBP: diastolic blood pressure; ^#^PP: pulse pressure.

**Table 3:** Univariable odds of pre-HT.

Variables	pre-HT	No pre-HT	OR	95% CI	*P* value
Malnutrition	10 (8)	52 (10.9)	0.71	0.33–1.4	0.22
Primary school	40 (32)	275 (57.5)	0.34	0.22–0.52	<0.00001
Private school	83 (66.4)	236 (49.4)	2.02	1.34–3.07	0.0004
Public school	42 (33.6)	242 (50.6)	0.49	0.32–0.74	0.0004
Secondary school	85 (68)	203 (42.5)	2.87	1.89–4.39	<0.000001
Migration	21 (16.8)	46 (9.6)	1.89	1.06–3.29	0.02
Obesity	11 (8.8)	14 (2.9)	3.19	1.37–7.27	0.006
Orphan	7 (5.6)	31 (6.5)	0.85	0.34–1.92	0.45
Promiscuity	47 (37.6)	228 (47.7)	0.66	0.43–0.98	0.02
Adolescents	84 (67.2)	224 (46.9)	2.31	1.53–3.53	<0.0001
HSL*	88 (70.4)	272 (57.1)	1.78	1.17–2.74	0.004
Overweight	16 (12.8)	31 (6.5)	2.11	1.09–3.98	0.019

*HSL: high socioeconomic level.

**Table 4:** Logistic regression to determine independent role of type of school, migration, obesity, HSL, and overweight on presence of pre-HT in schoolchildren.

Independent variables	*β* coefficient	Standard error	Wald *χ* ^2^	OR (95% CI)	*P* value
Private school (yes/no)	0.376	0.305	1.232	1.456 (0.8–2.65)	0.217
Secondary school (yes/no)	**0.902**	**0.230**	**3.921**	**2.466 (1.57–3.87)**	**0.0001**
Migration (yes/no)	**0.602**	**0.299**	**2.014**	**1.826 (1.01–3.28)**	**0.044**
Obesity (yes/no)	**1.221**	**0.430**	**2.835**	**3.391 (1.45–7.89)**	**0.0046**
HSL* (yes/no)	0.084	0.306	0.276	1.088 (0.59–1.98)	0.782
Overweight (yes/no)	**0.859**	**0.341**	**2.517**	**2.361 (1.2–4.61)**	**0.011**

*HSL: high socioeconomic level.

**Table 5:** Univariable odds of HT.

Variables	HT	No HT	OR	95% CI	*P* value
Malnutrition	3 (15)	59 (10.1)	1.56	0.44–5.49	0.24
Primary school	10 (50)	305 (52.4)	0.90	0.37–2.21	0.50
Private school	11 (55)	307 (52.7)	1.09	0.44–2.68	0.51
Public school	9 (45)	275 (47.3)	0.91	0.36–2.27	0.51
Secondary school	10 (50)	277 (47.6)	1.10	0.45–2.68	0.50
Migration	3 (15)	64 (11)	1.42	0.32–4.62	0.28
Obesity	4 (20)	21 (3.6)	6.67	2.05–21.71	0.004
Orphan	1 (5)	37 (6.4)	0.77	0.10–5.95	0.45
Promiscuity	5 (25)	269 (46.2)	0.38	0.13–1.08	0.047
Adolescents	13 (65)	294 (50.5)	1.81	0.71–4.62	0.147
HSL*	11 (55)	348 (60)	0.81	0.33–1.99	0.409
Overweight	6 (30)	41 (7)	5.65	2.06–15.49	0.002

*HSL: high socioeconomic level.

**Table 6:** Logistic regression to determine the independent role of type of school, obesity, orphans, adolescence, HSL, and overweight on presence of HT in schoolchildren.

Independent variables	*β* coefficient	Standard error	Wald *χ* ^2^	OR (95% CI)	*P* value
Private school (yes/no)	0.667	0.725	0.919	1.948 (0.46–8.08)	0.357
Secondary school (yes/no)	0.561	0.657	0.853	1.752 (0.48–6.36)	0.393
Migration (yes/no)	0.369	0.734	0.503	1.447 (0.34–6.11)	0.614
Obesity (yes/no)	**4.463**	**1.144**	**3.900**	**86.81 (9.21–818)**	**0.0001**
Orphans (yes/no)	−0.719	0.976	−0.737	0.486 (0.07–3.3)	0.461
Adolescents (yes/no)	0.981	0.692	1.418	2.669 (0.68–10)	0.156
HSL* (yes/no)	−0.723	0.666	−1.085	0.485 (0.13–1.79)	0.277
Overweight (yes/no)	**3.304**	**0.850**	**3.885**	**27.2 (5.14–144)**	**0.0001**

*HSL: high socioeconomic level.
